# Acceptance and commitment therapy on perceived stress and psychological flexibility of psychiatric nurses: a randomized control trial

**DOI:** 10.1186/s12912-021-00763-4

**Published:** 2021-11-30

**Authors:** Seyyed Arman Hosseini Zarvijani, Ladan Fattah moghaddam, Samaneh Parchebafieh

**Affiliations:** 1 Department of Psychiatric Nursing, Tehran University of Medical Sciences, Islamic Azad University, Tehran, Iran; 2 Department of Psychiatric Nursing, Faculty of Nursing and Midwifery, Tehran University of Medical Sciences, Islamic Azad University, Tehran, Iran; 3 Samaneh Parchebafieh, Department of Internal Surgery, Faculty of Nursing and Midwifery, Tehran University of Medical Sciences, Islamic Azad University, Tehran, Iran

**Keywords:** Acceptance and commitment therapy, Stress, Psychological flexibility, Psychiatric nurse

## Abstract

**Background:**

Nursing in psychiatric wards is considered a highly stressful career due to the type of patients and the problem of communicating with them. Finding appropriate solutions to overcome this stress can improve the general health of nurses and improve their quality of work. The aim was to investigate the impact of Acceptance and Commitment Therapy (ACT) on the perceived stress (PS) and psychological flexibility (PF) of nurses in psychiatric wards.

**Methods:**

A total of 70 nurses of Razi Psychiatric Center of Tehran were randomly selected and divided into two experimental and control groups of 35. In addition to routine interventions, the experimental group was provided with eight 2-h sessions of ACT training, whereas the control group only received routine interventions. Prior to the intervention sessions and a month after the last session, demographic information, PS scale, and Acceptance and Action Questionnaire (2nd Edition) were completed in both groups.

**Results:**

There was a significant difference regarding the PS level (*P* = 0.002) and PF (*P* = 0.001) in the control and experimental groups; the experimental group showed lower PS and higher PF.

**Conclusions:**

ACT can lead to reduced PS and improved PF, which can be considered as **a** solution to empower nurses working in psychiatric wards.

**Trial registration:**

This was registered in Iranian Registry of Clinical Trials (IRCT) (clinical trial code: IRCT20180506039557N1. Registered 2018-10-31. Retrospectively registered,

https://en.irct.ir/trial/31040

## Background

In modern societies, occupational stress and burnout are among the most important issues in a healthcare career [1]. Meanwhile, nursing is considered a high-stressed and challenging occupation due to its specialty, complexity, and the need to deal with emergency situations [2]. Issues such as communication with patients and their families, associating with doctors and other nurses, heavy workload, long hours, dissatisfaction with salary and fringe benefits, and the need to work on holidays tend to create stress in nurses [3]. Stress can lead to adverse consequences, and in case it exceeds the tolerance threshold of nurses, it can result in work-related outcomes such as increased absenteeism, reduced job satisfaction, lower productivity, and organizational commitment, as well as reduced quality of patient care [4]. Psychiatric wards are one of the most stressful centers among medical and educational centers [5], and nursing in these wards is considered a stressful occupation due to the type of patients and the problem of communicating with them. They continuously support and care for patients suffering from depression, anxiety, schizophrenia and bipolar, and personality disorders [6]. Working with other nurses and staff, communicating with patients, and high level of required skill and knowledge to work in psychiatric wards, heavy workload, the need for nurses to urgently respond to emergency situations, and heavy responsibility of caring for psychiatric wards with specific patients suffering from psychological problems are among the stress factors of this profession [7]. Nurses and staff are the major victims of aggressive behaviors from psychiatric patients as in general, 75% of psychiatric nurses are at least once exposed to aggression and assault from their patients, and those with lower scientific and practical abilities are more vulnerable [8, 9]. There is a significant positive correlation between exhaustion and psychological symptoms in nurses (e.g. depression, anxiety and stress) [10]. Durat et al. concluded that psychological inflexibility is a significant predictor of the high level of burnout, especially psychological exhaustion. Most nurses perceive thoughts, feelings, memories, physical feelings or internal experiences as “bad” or “unwanted”, and are experiencing more and more burnouts and psychological stress as a result of struggling to control or avoid them [11]. Burnout affects nurses’ work environment (e.g. reduced job performance, more absenteeism, etc.). Consequently, patients are also affected by worsening health care qaulity [12, 13]. A wide range of studies on nursing occupational stress indicates that individuals with high levels of one or more aspects of burnout have higher mean scores in perceived stress [14–16]. These results suggest that further research in this area is highly crucial and significant. Additionally, identifying major predictors of these types of consequences can make the development of systems, processes, and interventions related to these aspects more effective and help to provide efficient protection of individuals and organizations [17, 18].

The large changes in levels of stress indicated by health care providers may be indicated by individual psychological qualities, containing self-efficacy, coping skills, and motivation [19].

PF may be created within the contextual behavioral science and can be explained as a skill to act related to goals and values also in the presence of barring psychological experiences [20]. Thus, PF does not at first consider the symptoms of distress but instead the individual’s resilience and ability to cope well with the presence of distress [19]. It is the purpose of treatment in ACT [20], and to assess the PF to mediate outcomes in clinical trials in a variety of conditions such as chronic pain and anxiety [21, 22]. Acceptance and Commitment Therapy (ACT) is a practical solution to increasing mental health and reducing perceived stress [23]. This method is one of the third wave therapies of the cognitive-behavioral approach, whose main purpose is to develop PF; that is, choosing an action from a range of various choices that are more appropriate, rather imposition to act merely to avoid disturbing thoughts, emotions, memories or desires [24]. It has been discussed that PF has an important role in psychological health [25] and studies reported an incremental utility of PF over traditional measures of distress [26]. However, more research is required, such as studies that assess the associations between PF and general distress, as well as assessments of ability to differentiate between the constructs [27, 28]. Previous studies have considered the efficiency of ACT-based interventions within occupational health, and a meta-analysis by Öst et al. [29] reported it to be efficacious in targeting stress. These findings also indicate that PF may mediate changes in outcome [30, 31]. In a study on nursing students, suggested that ACT techniques may prevent the development of stress and burnout during nursing education [32]. Supporting findings on the effectiveness of ACT, compared to alternative methods, on problems such as depression, drug abuse, chronic pain, anxiety, psychosis, smoking, diabetes self-care, cancer adaptation, obsessive-compulsive disorder, and trichotillomania is rapidly growing [33, 34].

Although many studies have been performed on the effect of act on PF, none have been performed on psychiatric Nurses. In addition, this study was referred to Razi Psychiatric Hospital, the largest hospital in the Middle East, and all patients with psychotic disorders in Iran. No previous studies have been conducted on the act of the staff of this large hospital.

Considering the prevalence of stress among nurses, and assuming that promoting their PF may be effective in reducing their stress level, this study aims at determining the effects of ACT, as an educational method, on PS and PF of nurses engagged in psychiatric wards of Tehran Razi Psychiatric Center in 2018.

## Method

### Research hypothesis

The following research questions was addressed:

It could be argued that the hypothesis holding the effect of acceptance and commitment therapy on increasing the PF and reducing the PS of nurses is supported.

### Setting and study design

The present study was conducted at Tehran Razi Psychiatric Center with a research population consisting of psychiatric nurses working in the center in 2018. This study is considered as randomized control trial study in terms of data collection method, with a pretest-posttest design on experimental and control groups.

### Sample size and study sampling

To obtain the 95% confidence level and power of the test of 0.8% for the required sample size, it is hypothesized that the magnitude of the impact of ACT, as an educational method, on PS and PF of nurses engaged in psychiatric wards in the intervention group, in comparison to the control group, has at least the score of d = 0.7, in the following formula:

$$ 1+\mathrm{n}=\frac{{\left({Z}_{1-\frac{a}{2}}+{Z}_{1-\beta}\right)}^2}{d^{2.}} $$

**Fig. 1:**
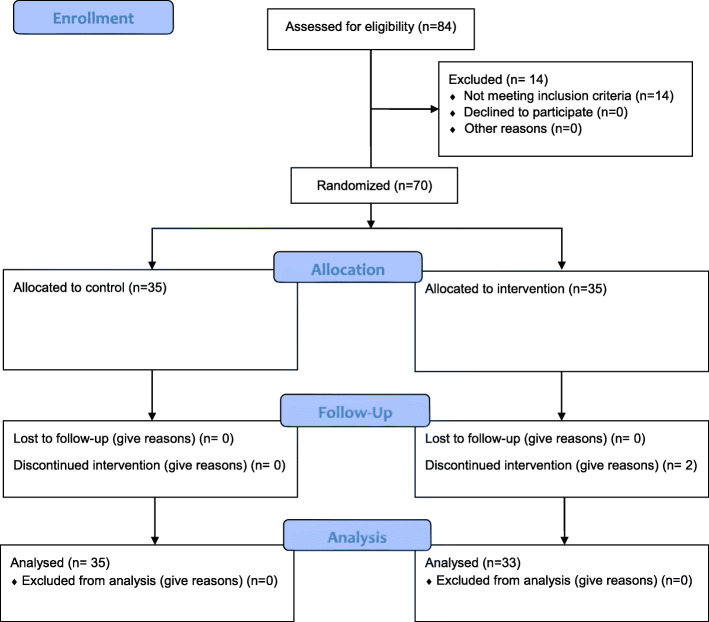
Flow diagram of the progress through the phases of two-group parallel randomized trial

We computed the sample size as 33 for the intervention group and 33 for the control group. It should be mentioned that according to the previous studies, the standard deviation of PS and PF were calculated to be 14.5 and with the probability of a sample dropout of 10% that was added to the sample size. Therefore, the sample size in each group was considered to be *n* = 35 (Fig. 1).

$$ n=\frac{2{\left(1.96+0.84\right)}^2}{0.7^2}=32+1 $$

### Random allocation

Samples were selected through stepwise random cluster sampling. The hospital where the study was carried out consisted of 23 wards. In each ward, a number of nurses were randomly selected based on the proportion of nurses to the sample size. Of the 84 nurses selected, fourteen were excluded due to lack of inclusion criteria, and 70 remaining nurses were each assigned a number and were randomly divided into experimental and control groups, each consisting of 35 participants. (Fig. 1).

### Inclusion and exclusion criteria

Inclusion criteria included having a bachelor’s or higher degree in nursing, at least 2 years of work experience in psychiatric wards, attending intervention sessions based on ACT for the first time, and no history of taking psychiatric drugs in the past and present. On the other hand, exclusion criteria included not completing the questionnaires, absenteeism in more than one intervention sessions, and the occurrence of stressful events during the study. Two of the participants in the experimental group were eliminated due to absence in more than one session.

### Intervention procedure

Routine interventions, the experimental group received ACT-based training according to Steven Hayes model in eight two-hour sessions conducted by an ACT therapist, whereas the control group merely received routine interventions such as stress control, life skills, and anger control workshops, which are normally held by the educational deputy of the center for the staff. After obtaining informed consent and providing sufficient explanations, prior to the intervention sessions, and a month after the last session, demographic information, PS scale, and Acceptance and Action (2nd Edition) questionnaire were completed by the participants. The control group participants completed the questionnaires in both stages (based on the schedule of the experimental group). The questionnaires were handed out to the participants after selecting the research sample (based on inclusion criteria) and providing sufficient explanation.

### Study instruments and measures

Three questionnaires were used to collect the required data:

#### Demographic information questionnaire

In this section, demographic information including age, sex, marital status, level of education, employment status and work duration was questioned.

#### Perceived stress scale (PSS)

The questionnaire was developed by Cohen et al. In 1983 and contained 14 questions to measure perceived general stress during the past month, as well as the thoughts and feelings about stressful events, and how to control, overcome, and cope with strain and experienced stresses [35]. The highest score that an individual can obtain in this questionnaire is 56, and the lowest score is zero. In case an individual obtains a score close to 56, it means that his or her PS has been high in the past month and vice versa. Cohen et al. (1983) measured the validity of PSS by calculating its correlation coefficient with semiotic dimensions, which was ranged from 0.52 to 0.76 [35], while Alsouni and Latif (2014) calculated its internal consistency by Cronbach’s alpha to be 0.74 [36]. In this study, the internal consistency of the questionnaire was calculated by Cronbach’s alpha to be 0.88, which is at an appropriate range.

#### Acceptance and action questionnaire- (AAQ-II)

This questionnaire was developed by Bond et al. (2007), and it is an edition of the original questionnaire (AAQ-I) created by Hayes (2000). The questionnaire assesses a construct associated with diversity, acceptance, experiential avoidance, and PF. It evaluates immobility and experiential avoidance, acceptance and action. AAQ-II items are rated on a 7-point Likert scale ranging from 1 (does not apply to me at all) to 7 (always applies to me). The highest score one can obtain in this questionnaire is 70 and the lowest score is 10. A high score indicates more experiential avoidance, while a low score reflects more acceptance and action. Bond et al. obtained the second version of Acceptance and Action Questionnaire using the Cronbach coefficient 0.81–0.88 [37]. In this research, the internal consistency of the questionnaire was calculated by Cronbach’s alpha to be 0.83.

### Statistical analysis

In order to unify the groups, the data regarding two participants in the control group were randomly selected and excluded from the calculations, and the resulting data with 35 individuals in experimental and control groups were analyzed using SPSS version 23 software. We carried out a one-way ANOVA to compare the relationship between demographic variables and PF and PS for the control and intervention groups. We also conducted Single-variable covariance for testing the effect of ACT on PF and PS of psychiatric nurses. The findings of Levene’s test on the homogeneity of error variances were not significant (*p* ≥ 0.05). In most studies in the statistical analysis considering the mean of two or several societies, the distribution of test statistics is addressed by assuming equal variances. Therefore, before applying the mean tests, it is necessary that the equality of variances in societies will be assessed by Levene’s Test. The Kolmogorov-Smirnov test represented the normality of data distribution (p ≥ 0.05).

## Results

Frequency and percentage of demographic characteristics including age range, sex, education and work experience, marital status and employment status of participants are presented in Table 1, and the mean score and standard deviation of pre-test and post-test variables in psychiatric nurses are shown in Table 2.

**Table 1:** Frequency and percentage of demographic characteristics of participants

Demographic Characteristics	Experimental Group		Control Group	
	Frequency	Percentage	Frequency	Percentage
**Age Range**				
Under 25	5	14.3	3	8.6
25–30	8	22.9	3	8.6
31–35	10	28.6	11	31.4
36–40	6	17.1	9	25.7
41 and over	6	17.1	9	25.7
**Gender**				
Male	16	45.7	17	48.6
Female	19	54.3	18	51.4
**Education**				
B.S	30	85.7	30	85.7
M.Sc	4	11.4	5	14.3
Ph.D.	1	2.9	0	0
**Work Experience**				
3–9 years	9	25.7	7	20
10–16 years	20	57.1	16	45.7
17–23 years	6	17.1	9	25.7
24–30 years	0	0	3	8.6
**Marital Status**				
Single	10	28.6	6	17.1
Married	25	71.4	28	80
Divorced	0	0	1	2.9
**Employment**				
Official Recruitment	17	48.6	19	54.3
Contract Worker	2	5.7	2	5.7
Company Worker	5	14.3	6	17.1
Project Worker	11	31.4	8	22.9

**Table 2:** Mean score and standard deviation of pre-test and post-test variables in psychiatric nurses in control and experimental groups

Variables	Experimental Group		Control Group	
	Pre-test Mean ± SD	Port-test Mean ± SD	Pre-test Mean ± SD	Port-test Mean ± SD
**PS**	23.42 ± 6.02	18.66 ± 5.44	23.36 ± 6.38	23.20 ± 5.70
**PF**	47.13 ± 9.42	55.49 ± 9.55	47.63 ± 7.56	47.56 ± 9.42

*PF* Psychological flexibility

*PS* Perceived stress

As can be seen from Tables 3 and 4, there is a significant difference between the level of PF and PS of nurses evaluated in the control and experimental group.

**Table 3:** One-way ANOVA table of the relationship between demographic variables and psychological flexibility and perceived stress

Variables	Groups	Pre-test		Post-test	
		significance level	F	significance level	F
Age * PF	Control	0.373	1.11	0.062	2.61
	Experimental	0.089	2.39	0.052	2.72
Age and * PS	Control	0.076	2.40	0.147	1.86
	Experimental	0.977	0.111	0.817	0.85
Education * PF	Control	0.868	0.033	0.960	0.003
	Experimental	0.942	0.060	0.977	0.023
Education * PS	Control	0.578	0.316	0.648	0.213
	Experimental	0.237	1.51	0.421	0.892
Sex * PF	Control	0.918	0.011	0.635	0.231
	Experimental	0.438	0.620	0.883	0.022
Sex * PS	Control	0.845	0.039	0.856	033.86
	Experimental	0.526	0.412	0.221	1.56
Work experience * PF	Control	0.926	1.51	0.244	1.47
	Experimental	0.136	1.15	0.066	3.01
Work experience* PS	Control	0.787	0.354	0.315	1.24
	Experimental	0.737	0.309	0.751	0.289
Marital Status * PF	Control	0.262	1.40	0.229	1.51
	Experimental	0.410	0.923	0.373	0.821
Marital Status * PS	Control	0.254	1.44	0.250	1.38
	Experimental	0.712	0.300	0.220	1.57
Employment status *PF	Control	0.272	1.37	0.825	0.300
	Experimental	0.142	1.97	0.448	0.914
Employment status * PS	Control	0.622	0.598	0.769	0.379
	Experimental	0.596	0.641	0.178	1.76

*PF* Psychological flexibility

*PS* Perceived stress

**Table 4:** Single-variable covariance test results related to the effect of ACT on psychological flexibility and perceived stress of psychiatric nurses

Test	Change Reference	Sum of Squares	Level of Freedom	Mean Squares	F	Level of Significance
**Variable**						
PF	Experimental group Pre-test	2720.31	1	2720.31	97.61	0.00
	Experimental group Post-test	1033.51	1	1033.51	37.8	0.00
PS	Experimental group Pre-test	1122.02	1	1122.02	103.24	0.00
	Experimental group Post-test	308.26	1	308.26	28.36	0.00*

*PF* Psychological flexibility

*PS* Perceived stress

## Discussion

The aim of the present study was to investigate the effect of acceptance and commitment therapy on PF and PS in psychiatric nurses. To the best of our knowledge, this is the first study to be conducted on psychiatric nurses. The results indicated that there was no significant relationship between demographic variables (age, sex, education, work duration, marital status, and employment status) and the mean score of flexibility and PS in experimental and control groups at both pretest and post-test levels. Based on the results of this study, it can be concluded that ACT has had a positive impact on improving the PF and reducing the PS of nurses. The results of this study are in line with many researchers, including Wersebe et al. and Frögéli et al. [23, 32]. In a study entitled “The link between stress, well-being, and psychosocial flexibility during an acceptance and commitment therapy self-help intervention “, conducted a study in Switzerland with 91 participants who experienced a high level of occupational stress, concluded that ACT helps reduce stress, promote well-being and increase PF, and increasing PF, in turn, improves well-being [23].

The PF model is considered as one of the most promising methods to cognitive behavioral therapy - ACT [38, 39]. Therefore, numerous evidence from systematic reviews have reported the positive effects of ACT for depression [39], anxiety [39] and subjective well-being [40] through improving PF among clinical and non-clinical people with small-to-medium effect sizes (Cohen’s d = 0.24–0.64) [39]. Also, the strong association between PF and prosociality in other studies demonstrates that prosociality is potentially malleable through ACT which may improve the mental health outcomes.

Mariola and colleagues reported that psychological approaches which considered the emotional regulation, cultivation of empathy, perspective-taking, gratitude, and compassion may improve the motivated prosocial behaviors of people [41]. Therefore, it is recommended for further ACT studies to investigate if addressing this malleable factor may nurture people with helping attitude and behaviour as an alternative way of coping. Also, recent research has indicated that even a very brief PF training, through using one experiential metaphorical exercise that aims to practice being in the present-moment awareness, may increase prosocial behavior [42].

In the present study, we found that a total increase in PF during the intervention was associated with a decrease in stress and an increase in well-being after the intervention. These results were in accordance with previous research in the therapy of stress among social workers [31], which reported that the variations in PF during intervention were related to decreases in stress during the intervention [32, 43].

Furthermore, mindfulness and reduction of PS and burnout were investigated by Frögéli et al. [32] and Markanday et al. [44]. Their results indicate that ACT was beneficial in improving psychological characteristics. The results of Brinkborg et al. [31] and Flexman et al. [45] showed that ACT reduced stress and increased general health. In another study, Swain et al. [46] found that ACT was effective on anxiety disorders.

Long-term high levels of stress typically may lead to disruptions in daily life, and studies have recommended that ACT changes the responses to stress which may cause to a decreasing of stress [47]. Our findings demonstrate that people with symptoms of stress may make a profit from a structured self-help intervention such as ours, which promoted changes in PF.

Well-being has significance for the individual, society, and the economy and also, our findings proved that PF is related to well-being. Evidence indicates that well-being is strongly connected to health care utilization, psychosocial adaptation and functioning [48, 49], and work productivity and that ACT interventions are positively associated with improving well-being [50, 51].

In this therapeutic approach, the therapist does not require the patient to accept the content of his thoughts, rather he encourages them to accept his thoughts as they are, not as his mental states. Thus, given that increasing distress is an inevitable part of normal human life, and avoiding a repeated experience often exacerbates distress and reduces the quality of life, then psychotherapy is necessarily required to help clients find ways to accept this distress. The major assumption of ACT is that a significant proportion of psychological distress is a normal component of human experience [52]. Hence, this method of treatment assists us in changing our relationship with our painful thoughts and feelings in a way that their influence and penetration in our lives is less severe. In fact, the purpose of ACT is not to directly change the references, but to help them to communicate with their experiences in different ways, and enable them to fully integrate into meaningful and value-based life.

### Limitation and strength of study

The present study had a few limitations which should be acknowledged. One of the main limitations of our study was randomization in one center and each group. Therefore, this randomization may have distorted the effects of the intervention. Another limitation was that, we ran the t-test analysis on the same material without using Bonferroni correction, so this can be a bias.

Our study had several strengths firstly, this study is the first study was conducted in Razi hospital in Iran and also the authors try to investigate acceptance and commitment therapy on these target patients. Secondly, this population has suffered deference psychiatric disorders who have not investigated before. Finally, the Rzai hospital is the beigest hospital in Middle East and it has great potential for health care services.

## Conclusion

It appears that training based on ACT techniques encourages individuals to frequently practice focusing on neutral stimuli and deliberate consciousness on the mind and body, and liberates them from engaging with threatening thoughts and concerns about relationships with others. That is, these techniques make the individuals aware of the present moment experiences and return their attention to the cognitive system, and a more comprehensive information processing causes concern, physiological stress, and strain in the individuals. In fact, commitment and acceptance therapy alters the mentality of nurses, and enables them to recognize their weaknesses and overcome them with the help of others. Therefore, they tend to contribute more to social communication, and this in turn instills positive thinking in the individual, and can reduce PS by improving individual’s morale and mental abilities, as well as their PF.

Due to the limited number of nurses, it was not possible for all nurses to participate in the sessions; therefore, each session was held twice a week. One of the limitations of this study was that it was impossible to track the results in long run due to the limited time of study and the fact that it was implemented in one psychiatric center. It is recommended that researches be conducted to compare the effect of ACT on PS and the PF of nurses in different wards, and to investigate the effect of ACT on PS of nurses using different measurement tools (e.g. interview, observation, and questionnaire). Also, comparing the impact of different methods of the third wave of psychotherapy and ACT on PS and PF of nurses can offer contributions to further improve the results of this study. Furthermore, qualitative research methods can further clarify the effectiveness of this interventional method.

## Data Availability

The datasets used and/or analyzed during the current study are available from the corresponding author on reasonable request.

## Abbreviations

ACT: Acceptance and commitment therapy
PS: Perceived stress
PF: Psychological flexibility
PSS: Perceived stress scale
AAQ-II: Acceptance and action questionnaire
